# Using aptamers to elucidate esophageal cancer clinical samples

**DOI:** 10.1038/srep18516

**Published:** 2015-12-21

**Authors:** Zhenxu Liu, Yi Lu, Ying Pu, Jun Liu, Bo Liu, Bo Yu, Ke Chen, Ting Fu, Chaoyong James Yang, Huixia Liu, Weihong Tan

**Affiliations:** 1 Xiangya Hospital, Central South University, Changsha, Hunan 410008, China; 2 Molecular Science and Biomedicine Laboratory, State Key Laboratory for Chemo/Bio-Sensing and Chemometrics, College of Chemistry and Chemical Engineering, College of Biology, and Collaborative Research Center of Molecular Engineering for Theranostics, Hunan University, Changsha, Hunan 410082, China; 3 State Key Laboratory for Physical Chemistry of Solid Surfaces, Key Laboratory for Chemical Biology of Fujian Province, Key Laboratory of Analytical Chemistry, and Department of Chemical Biology, College of Chemistry and Chemical Engineering, Xiamen University, Xiamen, 361005, China; 4 Department of Chemistry and Physiology and Functional Genomics, Center for Research at the Bio/Nano Interface, University Health Cancer Center, University of Florida, Gainesville, Florida 32611-7200, United States

## Abstract

The epithelial cell adhesion molecule (EpCAM) is closely correlated with the occurrence and development of various cancers of epithelial origin. This study tested, for the first time, the ability of EpCAM aptamer SYL3C to detect EpCAM expression in 170 cases of esophageal cancer (EC) and precancerous lesions, as well as 20 cases of EC series samples, using immunofluorescence imaging analysis. Corresponding antibodies were used as control. EpCAM overexpression was 98% in both esophageal squamous cell carcinoma (ESCC) and esophageal adenocarcinoma (EACA) and 100% in metastasis, but no EpCAM overexpression was detected in undifferentiated EC (UEC). Significant differences were noted among various stages of differentiation (p < 0.05) with the degree of differentiation inversely correlated with the expression of EpCAM. Overexpressed EpCAM was detected in severe dysplasia, but negative in mild to moderate dysplasia and benign esophageal lesions. In a competitive binding experiment, EpCAM aptamer generated a staining pattern similar to that of antibody, but the binding sites with EpCAM were different. Based on these results, it can be concluded that EpCAM is suitable for use as an EC biomarker, therapeutic target, and effective parameter for tumor transfer and prognosis evaluation by aptamer SYL3C staining.

Esophageal cancer (EC) is a high-risk cancer worldwide, but early diagnosis and treatment have substantially improved prognosis. The 5-year survival rate of patients who have undergone early surgery is 90% compared with only 10% in patients with metastasis, while untreated patients typically die within a year^1^. Because of the lack of an outer membrane in the esophagus, EC easily penetrates the esophageal wall, infiltrates into adjacent organs and metastasizes. By the time of initial diagnosis, unperceived tumor cell spread has occurred in most patients. In addition, no effective drug is available to eliminate minimal residual tumor cells after surgical resection of EC^2^. Therefore, new therapeutic targets are required to improve prognosis by the discovery and verification of EC-associated biomarkers.

The epithelial cell adhesion molecule (EpCAM) is a 40 kD transmembrane glycoprotein composed of three parts: the extracellular domain (EpEX), the intracellular domain (EpICD) and transmembrane domain. EpCAM is overexpressed in various cancers of epithelial origin and is closely related to carcinogenesis^3^,^4^,^5^,^6^. It has been reported that EpCAM overexpression in EC is correlated with poor prognosis. However, all these studies used antibody directed against EpEX antigen only, not EpICD, using a polyclonal antibody-based immunohistochemical (IHC) method. Moreover, the procedures reported were time-consuming with varied, and, thus, unreliable positive outcomes^7^,^8^,^9^,^10^,^11^,^12^. Furthermore, no studies have reported on EpCAM expression in undifferentiated EC (UEC).

Aptamers, consisting of ssRNA or ssDNA identified from an *in vitro* selection process called SELEX, have been termed chemical antibodies for their highly selective and specific target recognition and binding. Although aptamers are functionally analogous to antibodies, they have some advantages over antibodies, such as facile synthesis and chemical modification and lack of immunogenicity apart from their use in biosensors^13^,^14^,^15^,^16^,^17^,^18^. But the potential clinical applications of aptamers have not been fully explored. Our previous study showed that EpCAM aptamer SYL3C could specifically bind to EpCAM antigen in intestinal tissue, thus holding diagnostic value for colorectal cancer^19^. The present project was designed to test the feasibility of aptamer SYL3C as a molecular diagnostic/prognostic tool by evaluating its binding ability in the detection of EpCAM expression based on 170 cases of EC and precancerous lesions, as well as 20 cases of EC series samples (normal, borderline, cancer nest and metastasis), using immunofluorescence imaging analysis. Antibody-based IHC was employed as control.

## Results

### Specific staining of EC by aptamer SYL3C probe

All 20 cases of normal esophageal epithelium from the same patient with EC showed negative EpCAM expression by specific staining with aptamer SYL3C probe. However, sixty cases each, both ESCC and EACA, equally showed 98% overexpression of EpCAM by SYL3C, while all 20 cases of metastasis appeared as 100% overexpressed. In 20 cases of UEC, overexpressed EpCAM could not be found (Table 1). Ten cancer nests stained with aptamer SYL3C for both frozen tissue section and paraffin-embedded tissue sections showed similar results (Supplementary Fig. S1).

### Correlation of SYL3C immunoreactivity with various degrees of differentiation for EC

**Figure 1:**
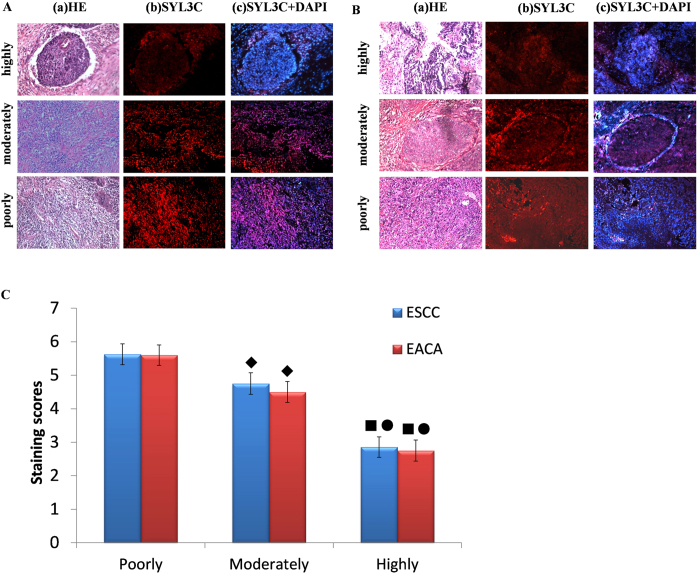
Correlation of SYL3C immunoreactivity with varying degrees of differentiation in both ESCC and EACA. (**A**) Specimens of highly, moderately and poorly differentiated ESCC. (**B**) Specimens of highly, moderately and poorly differentiated EACA: (a) H&E staining; (b) SYL3C-CY3 staining; (c) SYL3C-CY3 + DAPI staining. All pictures were taken under light microscopy with 200× magnification. (**C**) Scoring analysis of SYL3C-CY3 staining in ESCC and EACA at varying stages of differentiation. Tukey test showing distribution of total scores, as determined with SYL3C-CY3 staining in paraffin-embedded sections of EC representing different degrees of differentiation. No significant difference was found in fluorescent staining scores between ESCC and EACA with the same degree of differentiation (p > 0.05). However, as the degree of differentiation shifted downward from highly to poorly differentiated, fluorescence scoring shifted upward in both ESCC and EACA. (◼) Thus, the average score of highly differentiated EC (both ESCC and EACA) was lower than that of moderately differentiated EC (p < 0.05) (•) and also lower than that of poorly differentiated EC (p < 0.05). (♦) The average score of moderately differentiated EC, both ESCC and EACA, was lower than that of poorly differentiated EC (p < 0.05). ESCC: esophageal squamous cell; EACA: adenocarcinoma; poorly, poorly differentiated EC; moderately, moderately differentiated EC; highly, highly differentiated EC.

**Figure 2:**
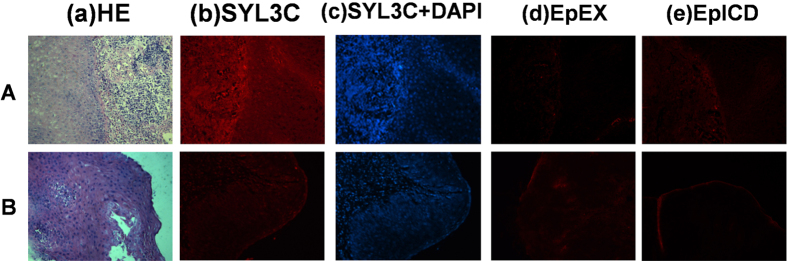
Immunostaining of UEC tissue sections with SYL3C-CY3 and EpCAM antibodies. (a) H&E staining; (b) SYL3C-CY3 staining; (c) SYL3C-CY3 + DAPI staining; (d) anti-EpEX staining and (e) anti-EpICD staining. In UEC, no overexpression of EpCAM was observed. (**A**), right side is necrotic tissue, while the left side is UEC. A mildly intense fluorescence signal was seen on the left side, but no signal was detected on the right side. Both anti-EpEX and anti-EpICD showed only background signal. (**B**), H&E staining confirmed positivity of UEC tissue, but neither SYL3C nor antibody staining detected it. All pictures were taken by light microscopy with 200× magnification. UEC: undifferentiated esophageal cancer.

All ESCC and EACA cases were divided into highly, moderately and poorly differentiated EC based on hematoxylin and eosin (H&E) staining. No significant difference in fluorescence staining scores was noted when the differentiation degree was the same between ESCC and EACA (p > 0.05). However, when the same tissue types (ESCC or EACA) showing different degrees of differentiation were compared with EpCAM staining by SYL3C probe, a significant difference among the various differentiation stages could be observed, such that a decreasing degree of differentiation correlated with increasing expression of EpCAM among ESCC or EACA (Fig. 1A,B). That is, while highly differentiated EC showed the weakest staining, poorly differentiated EC, inversely, showed the strongest staining. Therefore, as the degree of differentiation decreased, EpCAM showed a trend of increasing expression, and, as a result, significant difference was observed between EC tissue sections showing 3different degrees of cell differentiation (p < 0.05) (Fig. 1C). Moreover, out of 20 UEC cases, 30% showed mild positive EpCAM expression (6/20), but 70% of cases were negative (14/20) with no overexpression at all. As shown in Fig. 2(A), the right side was necrotic tissue, while the left side was UEC. Accordingly, a mildly intense fluorescence signal was seen on the left side, but no signal was detected on the right. Meanwhile, UEC sections of all 20 cases were also stained with antibodies (anti-EpEX and anti-EpICD, respectively), and the results were similar to those obtained with aptamer SYL3C staining [Fig. 2(d,e)].

### Series of immunostained EC tissue sections probed by EpCAM aptamer SYL3C and random sequences

**Figure 3:**
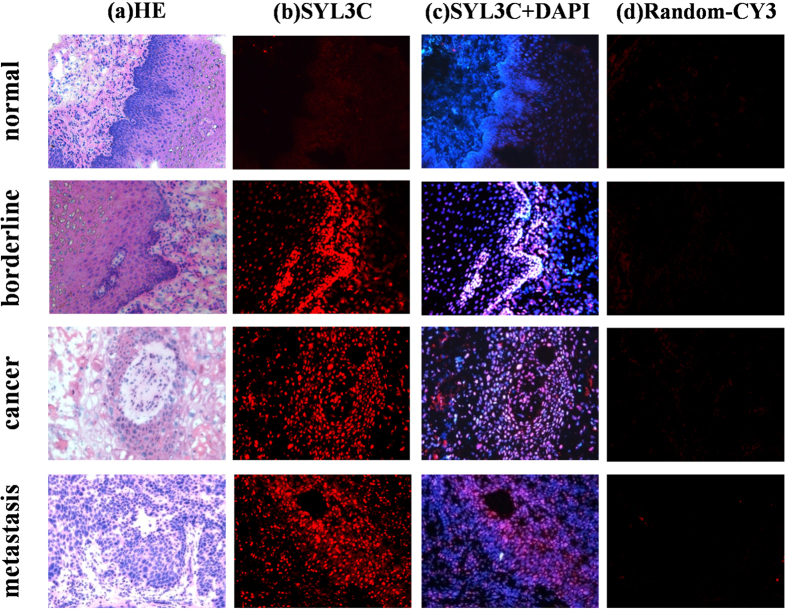
Series of immunostained EC tissue sections probed by EpCAM aptamer SYL3C-CY3. Normal, borderline, cancer nest and metastatic tissues from the same EC patient were made into a series of paraffin-embedded tissue sections. (**a**) H&E staining; (**b**) SYL3C-CY3 staining; (**c**) SYL3C-CY3 + DAPI staining; (**d**) Random sequences for negative control. The fluorescence signal of SYL3C-CY3-stained tissue sections increased from borderline to cancer nest to metastatic tissue, while normal tissue was negative. Images were obtained using light microscopy with 200× magnification.

For 20 cases, series specimens containing normal, borderline, cancer nest and metastatic tissue from the same patient were collected and probed by EpCAM aptamer SYL3C-CY3 and random sequences. Results showed negative EpCAM expression in normal esophageal tissue compared with EpCAM overexpression in all metastatic tissues. Furthermore, an increasing fluorescence intensity was observed from borderline to cancer nest to metastatic tissues, revealing significant difference in EpCAM expression between cancer nest with and without metastasis (p < 0.05). However, specimens in all 20 cases were negative upon probing by random sequences Fig. 3.

### Comparison between aptamer/antibody EpCAM expression and competitive binding experiment between EpCAM aptamer and antibody in EC tissue sections

**Figure 4:**
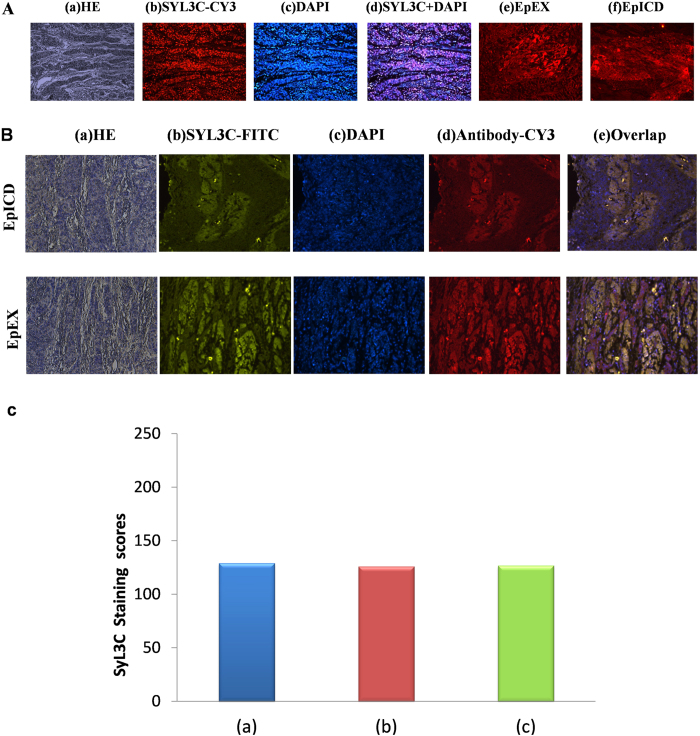
Comparison between aptamer/antibody EpCAM expression and competitive binding experiment between EpCAM aptamer and antibody in EC tissue sections. (**A**) Comparison between aptamer/antibody EpCAM expression in EC tissue. (a) H&E staining, (b) SYL3C-CY3 staining, (c) DAPI staining, (d) (SYL3C-CY3) + DAPI staining, (e) anti-EpEX staining, and (f) anti-EpICD staining. (**B**) Competitive binding experiment between EpCAM aptamer and antibodies (anti-EpEX or anti-EpICD) using the same slide from the same case shown in Fig. 4A. (a) H&E staining, (b) SYL3C-FITC staining (green), (**c**) DAPI staining (blue), (d) CY3-labeled EpCAM antibodies (red, upper row: anti-EpEX; lower row: anti-EpICD), and (e) overlapped by SYL3C-FITC + DAPI + antibody (yellow). (**C**).The fluorescence intensity of staining scores in competition test for both EpCAM aptamer and antibodies (anti-EpEX or anti-EpICD). (a) Fluorescence intensity from Fig. 4A when the specimens stained with aptamer SYL3C alone. (b,c) the fluorescence intensity (from Fig. 4B) of the same specimen when simultaneously stained with both EpCAM aptamer and antibodies (anti-EpEX or anti-EpICD).

EpCAM expression in stained sections was compared between aptamer and antibody. To accomplish this, forty cases of paraffin-embedded EC sections (20 cases each of ESCC and EACA) were stained with both aptamer SYL3C and antibodies (anti-EpEX and anti-EpICD, respectively). As shown in Fig. 4A, both aptamer and antibody staining showed similar results. However, taking a step forward, we then compared the binding patterns of EpCAM between aptamer and antibodies by performing a competitive binding experiment. Specimens were stained with both aptamer and antibodies (EpEX or EpICD) on the same slide from the same case as that shown in Fig. 4A. Specifically, slides were simultaneously stained with SYL3C-FITC [green, Fig. 4B(b)], DAPI [blue, Fig. 4B(c)], antibodies [red, Fig. 4B(d), upper row: anti-EpEX; lower row: anti-EpICD], and overlapped by SYL3C-FITC + DAPI + antibody [yellow, Fig. 4B(e)]. Results showed that specimens stained with either aptamer SYL3C or antibodies (EpEX and EpICD, respectively) exhibited the same fluorescence intensity (Fig. 4A). Even when the same specimen was simultaneously stained with both EpCAM aptamer and antibody, the aptamer fluorescence intensity was equivalent to that of the antibodies (Fig. 4B). Finally, we compared the binding ability of aptamer both aptamer separately and stained with both EpCAM aptamer and antibody, simultaneously, with the EpCAM antigen. It was found that the fluorescence intensity of aptamer stained alone is similar to that of aptamer stained with both aptamer and antibody in the same slide. (Fig. 4C). While EpCAM aptamer generated an EpCAM staining pattern similar to that of antibody, as noted above, results of this experiment show that its binding sites with EpCAM antigen were different. Therefore, no competition was observed between aptamer and antibody in the same EC tissue section.

### SYL3C immunostaining analysis in dysplasia and benign lesion of esophagus

**Figure 5:**
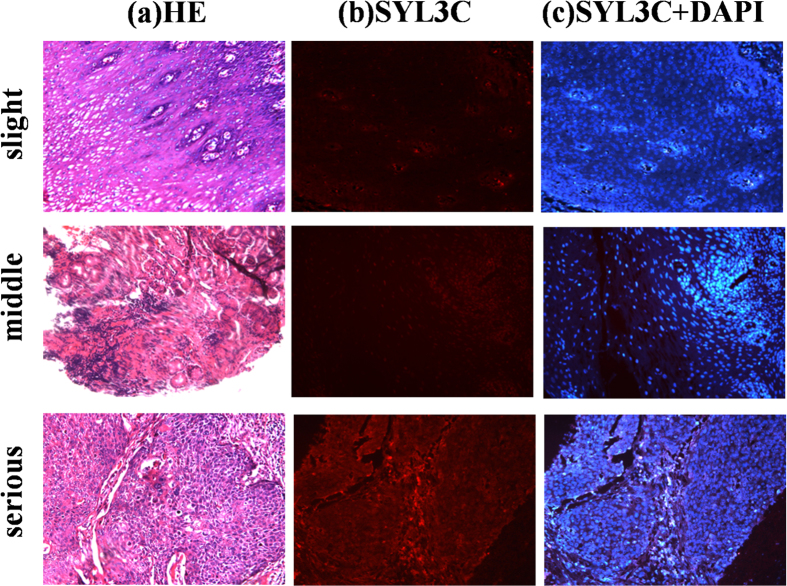
Specific immunostaining of esophageal dysplasia tissue sections by EpCAM aptamer probe SYL3C-CY3. (**a**) H&E staining, (**b**) SYL3C-CY3 staining and (**c**) SYL3C-CY3 + DAPI staining. No fluorescence signal was noted for either slight or moderate dysplasia cases, but overexpressed EpCAM was observed in all cases of severe hyperplasia. Images were obtained using light microscopy with 200× magnification.

**Figure 6:**
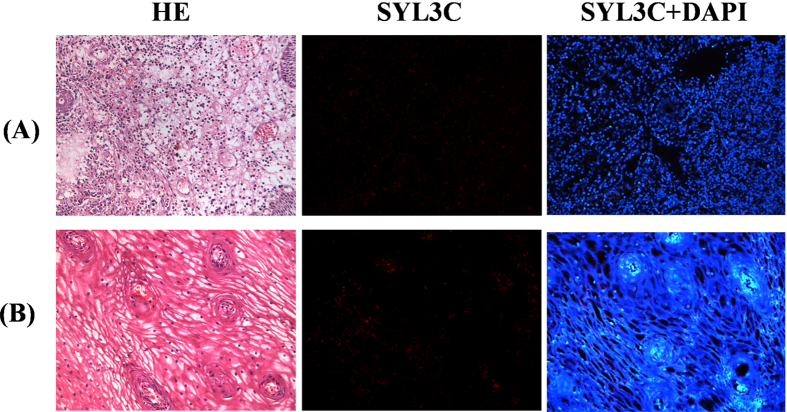
No cross-reaction of EpCAM aptamer probe to esophageal benign lesions. (**A**) esophageal chronic inflammation, (**B**) squamous metaplasia of esophagus. H&E staining confirmed all pathological diagnoses. Images were obtained using light microscopy with 200× magnification.

A total of 30 cases of esophageal dysplasia (10 cases each showing slight, moderate and severe dysplasia) and 20 cases of benign lesion of esophagus (10 cases each of esophageal chronic inflammation and squamous metaplasia of the esophagus) were probed with SYL3C. Cases of severe dysplasia showed 100% overexpression compared with slight and moderate cases and benign lesion of esophagus, all of which presented negative EpCAM expression (Figs 5 and 6).

## Discussion

Thus far, prognosis of EC remains very poor, even after curative resection in locally advanced EC^20^. Effective adjuvant therapy following surgery has been regarded as an effective, but challenging, strategy for improving the prognosis of EC.

Historically, EC can be divided mainly into ESCC and EACA, the former accounting for most cases of EC. In Western countries, the incidence of these two types of cancer has been comparable in recent years^21^. TNM staging of EC is an important prognostic factor, but it can only be assessed reliably after surgery. Consequently, it is necessary to find a new and reliable biomarker able to signal the degree of tumorigenesis, allowing clinicians to determine, preoperatively, which patients would be best served by adjuvant treatment.

Aptamers represent a new class of molecular tool for cancer diagnosis, but their clinical applications are still limited. Here, we have first demonstrated the pathological significance of EpCAM overexpression in EC by using an EpCAM-targeted aptamer termed SYL3C. Results showed that overexpressed EpCAM was equally scored in both ESCC and EACA at 98% and almost 100% within metastatic tissues. Generally, a decreasing degree of differentiation is most correlated with increasing degree of malignancy and worse prognosis in cancers^22^. Therefore, in the present study, EpCAM expression was compared among highly, moderately and poorly differentiated EC, and, as expected, results showed an inverse relationship between differentiation and EpCAM expression, in that the lower the degree of differentiation, the stronger the EpCAM expression. Therefore, while EpCAM signal intensity is inversely associated with degree of differentiation, it is positively correlated with the degree of malignancy among EACA and ESCC except UEC.

Specimens from 20 cases of UEC, the most aggressive type of EC, were stained by aptamer SYL3C, and no overexpressed EpCAM could be found, with only 30% mild EpCAM expression. We asked what circumstances would account for this interesting result, and we looked to the literature for clues. In undifferentiated thyroid cancer, Ralhan *et al.*^23^ reported that negative expression of EpCAM in undifferentiated thyroid cancer resulted from the loss of membrane EpEX through hydrolysis and the shift of EpICD to nucleus, but the gain of EpICD expression within the cells, indicating poor prognosis. However, in our study, the slides of UEC stained by not only aptamer SYL3C but also antibodies (EpEX and EpICD) gave identical results; a very low EpCAM signal was observed in UEC, which cannot be explained by hydrolytic cleavage or the shift of EpICD to the nucleus. So far, the exact mechanism of action of EpCAM contributing to the malignant potential of tumor cells is not fully understood, and therefore further exploration is needed.

The major reason for cancer-related death is the development of metastasis. As such, the biomedical importance of new metastasis-related biomarker discovery and verification cannot be underestimated. In this study, significant difference of EpCAM overexpression was revealed between cancer nest with and without metastasis and 100% overexpression of EpCAM in metastatic tissues probed by SYL3C. These results suggest that EpCAM could be used as a biomarker to evaluate the state of metastasis and as a promising molecular therapeutic target for EC. Based on these results, we concluded that aptamer SYL3C could also play a pivotal role in detecting EpCAM and delivering novel drugs.

Anti-EpCAM antibody-based IHC is the most widely used method for detection of EpCAM^24^. However, our comparison studies showed the simplicity of the aptamer-based tissue imaging method. The aptamer method needs to be performed only once, while to achieve a similar overexpression rate with the antibody-based method, different antibodies, including anti-EpEX and anti-EpICD + secondary antibody are needed. Therefore, this study demonstrates that 1) EpCAM-specific aptamer SYL3C can recognize EpCAM antigen from EC with high affinity, 2) the method is simple, and 3) the imaging is intuitive and easy to perform. These results suggest that the EpCAM aptamer can replace antibodies, in particular those used for the detection of EpCAM expression in EC.

To explore the difference of EpCAM antigen binding mode between antibody and aptamer, we, for the first time, carried out a competitive binding experiment by using the same EC tissue slide. Our results demonstrated that aptamer and antibody generated the same EpCAM staining pattern, but at different binding sites, without competing with one another in the same tissue. This evidence provides a theoretical basis for targeted therapy combining both aptamer and antibody with dual-phase drug loading.

The prevention of EC depends on the early detection of precancerous lesions and surgical intervention. In the case of severe dysplasia, which is a precancerous lesion, endoscopic therapy should be performed once diagnosis has been confirmed. Our results indicated that EpCAM is overexpressed in severe dysplasia, but negative in mild to moderate cases and in benign esophageal lesions. Therefore, by using SYL3C to stain esophageal lesions, we should be able to detect precancerous lesions and distinguish benign from malignant lesions.

Here we present the first comprehensive study of EpCAM expression in various types of EC in the context of aptamer-based staining. Indeed, a number of comparisons were made, and morphological H&E staining was performed as control. We noticed that normal epithelial tissues from the same cancer patient with overexpressed EpCAM were negative for SYL3C staining and that cancer tissue showed strong positive staining of SYL3C, while the random sequence was negative for EpCAM expression. Comparable results were obtained for frozen tissue sections and paraffin-embedded tissue sections stained with SYL3C. Furthermore, when comparing antibody- and aptamer-based staining, consistent results were obtained, indicating that EpCAM could be used as a suitable biomarker for EC diagnosis, as well as a therapeutic target. This is the first time aptamer technology has been employed for clinical diagnosis on a large scale. The results suggest that aptamer-based detection could provide a promising diagnostic and prognostic tool and that aptamer SYL3C represents an equally promising class of delivery vehicles able to target EC.

## Materials and Methods

### Patients and tissue specimens

A total of 190 paraffin-embedded esophageal lesions were collected and diagnosed at the Department of Pathology of Xiangya Hospital (Changsha, Hunan, CN) from January 2001 to November 2014, in accordance with the 2010 4th edition of the World Health Organization classification of tumors of the digestive system. These specimens reflected 140 cases of EC, including 20 cases each of highly, moderately and poorly differentiated esophageal squamous cell carcinoma (ESCC) and esophageal adenocarcinoma (EACA); 20 cases of UEC; 30 cases of dysplasia (10 cases each of mild, moderate and severe dysplasia); and 20 cases of esophageal benign lesion, including 10 cases each of chronic esophagitis and squamous hyperplasia. Meanwhile, 20 EC cases representing a series of fresh tissue samples, including normal esophageal tissue, borderline between normal and cancer nest, cancer nest tissue, as well as metastasis, were collected from the operating room of Xiangya Hospital from January 2013 to October 2014. Ten cancer nest of these cases were made into three frozen tissue sections for H&E stain, SYL3C and random sequence staining, and all of the 20 EC cases were freshly paraffin-embedded. All of the sections were stained with H&E stain. These sections were separately stained with EpCAM aptamer and random aptamer sequence, as well as the control anti-EpEX and anti-EpICD antibodies. All slides were reviewed by pathologists prior to further experiments.

### Paraffin-embedded tissue sections

The tissues were fixed in 10% formalin for 24 h at room temperature (RT), then washed and embedded in paraffin blocks. After slicing, the sections were mounted on slides, air dried for 30 min and incubated overnight at 60 °C. The production process of frozen tissue sections was described previously^19^.

### Fluorescence-labeled EpCAM aptamer(SYL3C-CY3) and antibodies

The oligonucleotide DNA aptamer SYL3C probe labeled with Cy3 (SYL3C-Cy3, 48 bp) was synthesized with the following sequence: 5′-CAC TAC AGA GGT TGC GTC TGT CCC ACG TTG TCA TGG GGG GTT GGC CTG-(PEG)3-Cy3-3′^25^. An aptamer random sequence (5′-rN-3′, 48 bp) was also generated (Sangon Biotech, Shanghai, CN). 4′, 6-diamidino-2-phenylindole dihydrochloride (DAPI) (Beyotime Institute of Biotechnology, Shanghai, CN) was used to label the nucleus. Anti-human EpCAM mouse monoclonal antibody MOC-31 (Catalog No. ab187270) recognizes EpEX antigen, and the secondary antibody was also labeled with Cy3 (Catalog No. ab97035). Rabbit monoclonal antibody E144 (Catalog No. ab32392) recognizes EpICD antigen, and the secondary antibody was also labeled with Cy3 (Catalog No. ab97075). All antibodies were from Abcam, Cambridge, MA.

### Deparaffinization and antigen retrieval of paraffin-embedded sections

Tissue sections were deparaffinized three times in xylene and washed with 100% ethanol, followed by 95% ethanol and 75% ethanol, then blocked by 3% perhydrol for 2 min. For antigen retrieval, tissue sections were boiled in sodium citrate buffer (pH 6.2) for 25 min, followed by incubation in binding buffer (PBS with 20% fetal calf serum and 1 mM DNA sodium salt from calf thymus) at RT for 1 h.

### Staining of paraffin-embedded tissue sections with SYL3C-CY3 and CY3-labeled anti-EpEX and anti-EpICD antibodies

The sections were deparaffinized and antigen retrieved before and after two washes with binding buffer. For aptamer staining, sections were incubated with 1 μM random sequence for 30 min and incubated with 250 nM SYL3C-CY3 for 1 h. Sections were then washed three times with binding buffer, sealed, and 5 μL DAPI added for 3 min. The fluorescence signal was observed using fluorescence microscopy.For antibody staining, sections were incubated with anti-EpEX or anti-EpICD (1:100 dilution) at 37 °C for 30 min, respectively. After washing with PBS, secondary antibody labeled with CY3 (1:200 dilution) was added and further incubated for 30 min at 37 °C, followed by washing in PBS. Five μL DAPI was added on tissue slides and stained for 3 min, followed by washing in distilled water before viewing under fluorescence microscopy. All steps were performed in the dark.

### Competitive binding experiment between aptamer and antibody

Tissue sections were deparaffinized, and antigen retrieval was carried out as above. The same section was blocked with 1 μM random sequence and then incubated with primary antibody (anti-EpEX or anti-EpICD, 1:100 dilution) at 37 °C for 30 min. After three consecutive washings, the section was incubated with 250 nM SYL3C-FITC and secondary antibody labeled with CY3 (1:200 dilution) at the same time for 30 min in 37 °C. Again, 5 μL DAPI was added for 3 min, followed by another round of washing with PBS 3 times. After a final washing in distilled water, sections were examined by fluorescence microscopy. All steps were performed in the dark.

### Evaluation of immunostained sections for both aptamer and antibody and statistical analysis

The immunostained sections were evaluated in five areas as described^23^. Two professional pathologists blinded to the clinical outcome were employed. Tissue sections were scored as positive if epithelial cells showed immunopositivity in the plasma membrane, cytoplasm, and/or nucleus, according to the following standard: 0, <10% cells; 1, 10–30% cells; 2, 30–50% cells; 3, 50–70% cells; and 4, >70% cells. Also, sections were scored semi-quantitatively based on fluorescence signal intensity, as follows: 0, none; 1, mild; 2, moderate; and 3, intense. Finally, a total score (ranging from 0 to 7) was obtained^23^,^26^, and a total score more than 3 for each section was defined as overexpressed. Immunofluorescence scoring data were verified using the Visiopharm Integrator System (Visiopharm, Horsholm, Denmark) and then subjected to Turkey’s HSD (honest significant difference) test and, finally, statistical analysis using SPSS13.0 software (SPSS Inc., Chicago, IL). For the competitive binding experiments, fluorescent intensity was analyzed as described previously^27^.

### Medical Ethics

All specimens were obtained from Xiangya Hospital (Changsha, Hunan, CN) in compliance with a protocol approved by the Institutional Review Board of Central South University of Xiangya Hospital.

## Additional Information

**How to cite this article**: Liu, Z. *et al.* Using aptamers to elucidate esophageal cancer clinical samples. *Sci. Rep.* **5**, 18516; doi: 10.1038/srep18516 (2015).

## Supplementary Material

Supplementary Information

## Figures and Tables

**Table 1:** EpCAM expression in esophageal cancer as detected by aptamer SYL3C staining.

Variable	ESCC	EACA	UEC	Metastasis	Normal
n	60	60	20	20	20
Overexpression(%)	98%	98%	^*^30%	100%	0%

EpCAM overexpression as defined by Ralhan R^23^.

*mild EpCAM expression.

ESCC, esophageal squamous cell carcinoma; EACA, esophageal adenocarcinoma; UEC, undifferentiatedesophageal cancer.
