# Supplementary material for: Using aptamers to elucidate esophageal cancer clinical samples
Source: Sci Rep. 2015 Dec 21;5:18516. doi: 10.1038/srep18516 (PMC4685197; doi:10.1038/srep18516)
Supplement: Supplementary Information [file srep18516-s1.pdf]

Using aptamers to elucidate esophageal cancer clinical samples

Zhenxu Liu, Yi Lu, Ying Pu, Jun Liu, Bo Liu, Bo Yu, Ke Chen, Ting Fu, Chaoyong James Yang, Huixia Liu ✉, and Weihong Tan ✉

Supplementary Figure

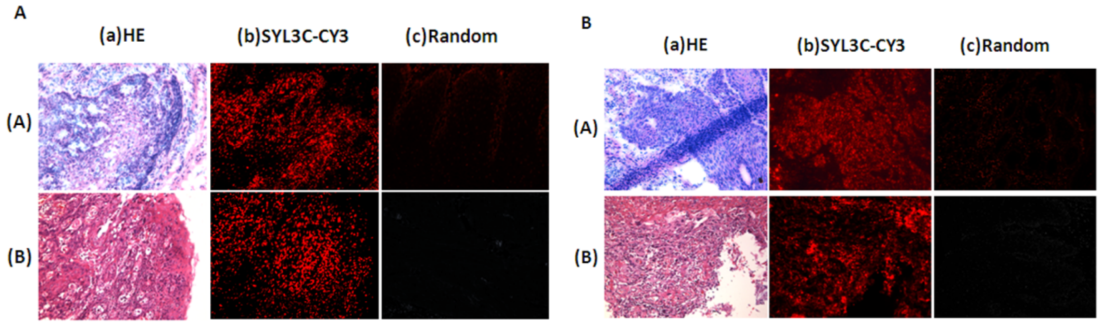

Supplementary Figure S1. Comparing SYL3C staining in EC between frozen tissue sections and paraffin-embedded tissue sections. A. Specimen of ESCC; B. Specimen EACA; (A) frozen tissue section; (B) paraffin-embedded tissue section; (a) H&E staining; (b) SYL3C-CY3 staining; (c) random sequence staining. All pictures were taken under light microscopy with 200× magnification.

ESCC: esophageal squamous cell carcinoma; EACA : esophageal adenocarcinoma;
